# Synthetic RORγ agonists regulate multiple pathways to enhance antitumor immunity

**DOI:** 10.1080/2162402X.2016.1254854

**Published:** 2016-11-04

**Authors:** Xiao Hu, Xikui Liu, Jacques Moisan, Yahong Wang, Charles A. Lesch, Chauncey Spooner, Rodney W. Morgan, Elizabeth M. Zawidzka, David Mertz, Dick Bousley, Kinga Majchrzak, Ilona Kryczek, Clarke Taylor, Chad Van Huis, Don Skalitzky, Alexander Hurd, Thomas D. Aicher, Peter L. Toogood, Gary D. Glick, Chrystal M. Paulos, Weiping Zou, Laura L. Carter

**Affiliations:** a Lycera Corp, Ann Arbor, MI, USA; b Medical University of South Carolina, Hollings Cancer Center, Charleston, SC, USA; c University of Michigan, School of Medicine, Ann Arbor, MI, USA

**Keywords:** Adoptive cell therapy, co-inhibitory receptors, co-stimulatory receptors, immunotherapy, PD-1, RORγ, Tc17, Th17

## Abstract

RORγt is the key transcription factor controlling the development and function of CD4^+^ Th17 and CD8^+^ Tc17 cells. Across a range of human tumors, about 15% of the CD4^+^ T cell fraction in tumor-infiltrating lymphocytes are RORγ+ cells. To evaluate the role of RORγ in antitumor immunity, we have identified synthetic, small molecule agonists that selectively activate RORγ to a greater extent than the endogenous agonist desmosterol. These RORγ agonists enhance effector function of Type 17 cells by increasing the production of cytokines/chemokines such as IL-17A and GM-CSF, augmenting expression of co-stimulatory receptors like CD137, CD226, and improving survival and cytotoxic activity. RORγ agonists also attenuate immunosuppressive mechanisms by curtailing Treg formation, diminishing CD39 and CD73 expression, and decreasing levels of co-inhibitory receptors including PD-1 and TIGIT on tumor-reactive lymphocytes. The effects of RORγ agonists were not observed in RORγ−/− T cells, underscoring the selective on-target activity of the compounds. *In vitro* treatment of tumor-specific T cells with RORγ agonists, followed by adoptive transfer to tumor-bearing mice is highly effective at controlling tumor growth while improving T cell survival and maintaining enhanced IL-17A and reduced PD-1 *in vivo*. The *in vitro* effects of RORγ agonists translate into single agent, immune system-dependent, antitumor efficacy when compounds are administered orally in syngeneic tumor models. RORγ agonists integrate multiple antitumor mechanisms into a single therapeutic that both increases immune activation and decreases immune suppression resulting in robust inhibition of tumor growth. Thus, RORγ agonists represent a novel immunotherapy approach for cancer.

## Introduction

RORγt, as a master transcription factor, plays a key role in the differentiation and maintenance of Type 17 effector subsets of CD4^+^ (Th17) and CD8^+^ (Tc17) T cells, and is also pivotal in the differentiation of IL-17-expressing innate immune cell subpopulations (e.g., subsets of innate lymphoid cells, NK cells, γδT cells, and iNK T cells).^1,2^ These cells are critical for mediating immune responses against fungi, other microbes and cancer cells and are distinguished from other subsets by their production of cytokines IL-17A, IL-17F, GM-CSF and IL-22, and chemokine CCL20.^2-5^ In addition, RORγt plays a critical role in the generation of mature T cells with diverse TCR for antigen recognition by controlling the survival of thymocytes and TCR recombination at the CD4^+^CD8^+^ double positive stage.^6,7^ After T cells mature and emigrate from the thymus, only a small fraction of peripheral blood mononuclear cells (PBMCs) from healthy donors express RORγt. However, this transcription factor can be induced by cytokines such as TGFβ and IL-6.

Existing literature data suggest that Th17 and Tc17 cells can mediate potent and durable tumor growth inhibition when transferred to tumor-bearing animals and their hallmark cytokines such as IL-17A and GM-CSF are associated with improved antitumor effects in some cancers.^8-11^ Human Th17 cells stimulated with ICOS and re-directed with a chimeric antigen receptor (CAR) construct showed persistent tumor killing activity in mice implanted with human mesothelioma.^12,13^ However, both anti- and pro-tumors effects have been reported for IL-17A.^14^ These seemingly inconsistent data may derive from the multi-faceted immune responses associated with Type 17 effector cells while IL-17A, as a single cytokine, may manifest anti- or pro-tumor effects depending on the tumor environment or tumor type.

In this report, we show that activation of RORγ with small molecule, synthetic agonists enhances T cell effector functions and decreases immune suppressive mechanisms, leading to improved antitumor efficacy in adoptive cell therapy (ACT) models and in syngeneic murine tumor models. Thus, RORγ agonists represent a novel approach for next generation cancer immunotherapies.

## Results

### Identification of synthetic RORγ agonists

**Figure 1.**
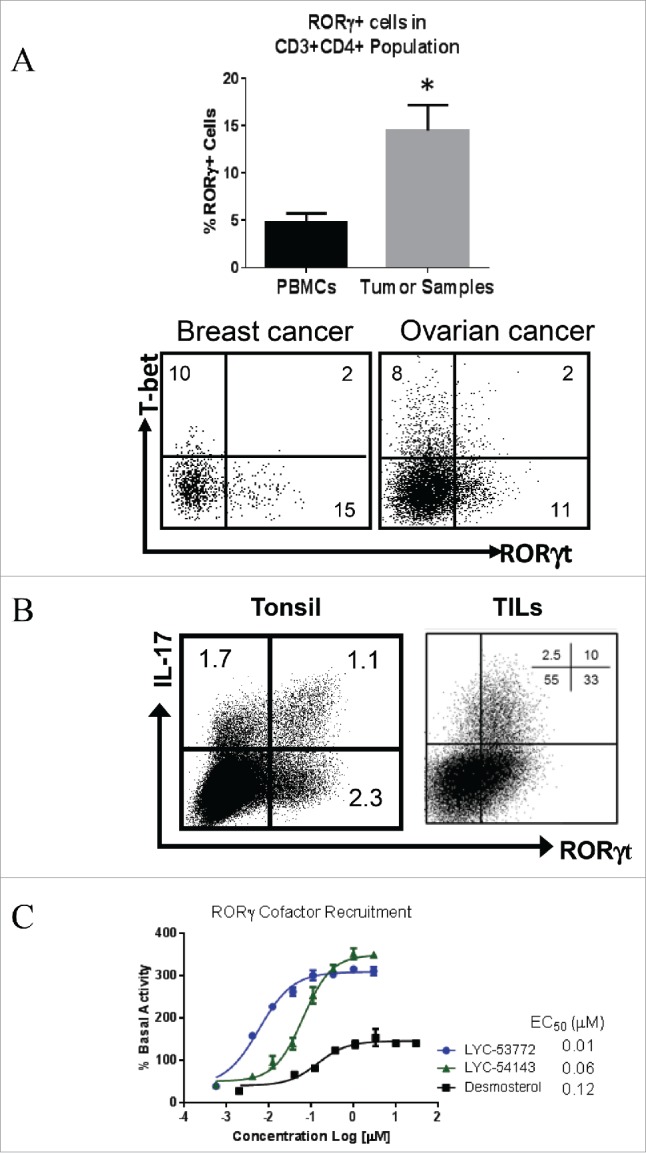
Expression of RORγ in human tumors and identification of RORγ agonists. (A) RORγ+ T cells are present in significant fractions in TILs from various tumor types. Total of 14 tumor samples from colon, ovarian, lung, breast and head and neck cancers. Cells were gated on CD45^+^CD3^+^CD4^+^. *p = 0.03 tumor vs. PBMCs. Unpaired, two-tailed *t*-test. Bottom. Flow graph shows an example of staining. (B) Co-staining of IL-17A and RORγt shows partial overlap of RORγ and IL-17A expressing cells. (C) A TR-FRET-based assay was used to show that RORγ agonists increase co-activator recruitment. Each data point represents mean ± standard deviation (SD) of biological triplicates.

Administration of Type 17 immune cells, especially CD8^+^ Tc17 and CD4^+^ Th17 cells generates durable antitumor immunity in ACT settings.^8-10^ This efficacy is associated with enhanced persistence of antitumor cells and robust *in vivo* cytotoxic activity.^10,15^ To assess the prevalence of these cells in human cancers, we evaluated the expression of the Type 17 master transcription factor, RORγ, in tumor-infiltrating lymphocytes (TILs) and PBMCs from cancer patients. RORγ^+^ T cells are present at significantly higher frequencies in tumors compared to blood, suggesting that the tumor microenvironment recruits these cells or promotes their generation (Fig. 1A). The percentage of RORγ^+^ T cells is similar to that of cells expressing T-bet, the hallmark transcription factor of Th1 cells (Fig. 1A). Interestingly, only a fraction of human T cells from either tumor or tonsil co-expresses both RORγt and IL-17A, while a significant fraction expresses either IL-17A or RORγ alone (Fig. 1B). These data suggest that RORγ and IL-17A may play distinct roles in antitumor immunity.

Given the presence of RORγ^+^ cells in human tumors and the antitumor effects of Type 17 T cells reported in animal models, we sought to evaluate whether activating RORγ with synthetic agonists would enhance Type 17 T cell differentiation and function and improve their antitumor activity. We identified a series of synthetic agonists of RORγ using a time resolved-fluorescence resonance energy transfer (TR-FRET) assay. This assay detects the ability of a synthetic compound to enhance recruitment of co-activator steroid receptor co-activator 1 (SRC1) to the ligand-binding domain of RORγ and was previously used to identify the cholesterol synthesis precursor desmosterol and desmosterol-sulfate as endogenous RORγ agonists.^16^

Fig. 1C shows that two synthetic compounds, LYC-53772 and LYC-54143, enhance SRC1 recruitment. Both compounds were more potent and induced higher co-activator recruitment than the endogenous agonist desmosterol. These compounds were further characterized in a cellular reporter assay using a Gal4-RORγ fusion construct.^16^ To enhance the assay window, the basal activity of RORγ was lowered with a known antagonist, ursolic acid. Under this assay condition, desmosterol did not enhance the reporter activity over the basal activity of RORγ (Fig. S1). In contrast, the two synthetic agonists robustly enhanced the reporter to about 150% of the basal RORγ activity, confirming that they induce stronger activation than the endogenous agonists. LYC-53772 and LYC-54143 are potent RORγ agonists with EC_50_s of 0.6 ± 0.1 and 0.2 ± 0.1 μM, respectively, in this assay. In addition, neither compound activated closely related nuclear receptors including RORα and RORβ (Table S1), suggesting that they selectively activate RORγ.

### Effects of synthetic RORγ agonists on Th17, Tc17, and Treg differentiation

**Figure 2.**
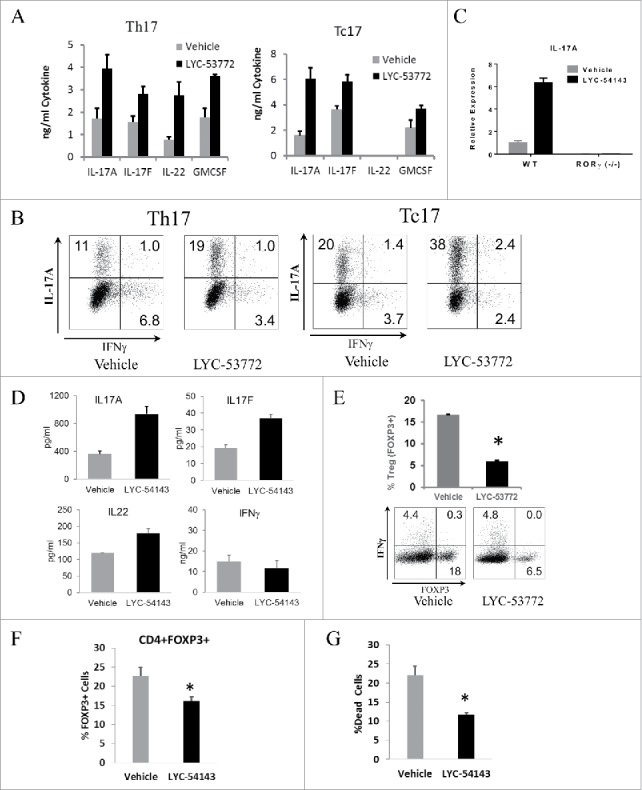
RORγ agonists enhance Type 17 differentiation and cytokine production. (A) RORγ agonist LYC-53772 increased Type 17 cytokines when added to both Th17 and Tc17 differentiation cultures. LYC-53772 vs. vehicle, *p* < 0.007 for Th17 and *p* < 0.03 for Th17 and Tc17, respectively. Data represent mean ± SD of biological quadruplicates. (B) LYC-53772 increased the percentage of CD4^+^IL-17A^+^ (Th17, left) and CD8^+^IL-17A^+^ (Tc17, right) cells with minimal effects on their production of IFNγ (representative flow graph). (C) Elevated IL-17A expression was dependent on RORγ. IL-17A mRNA was assayed by qPCR. Data represent mean ± SD of biological triplicates. (D) RORγ agonist LYC-54143 increased cytokine production in human PBMCs polarized under Type 17 conditions. LYC-54143 vs. vehicle, *p* < 0.03 for IL-17A, IL-17F, or IL-22, *p* = 0.5 for IFNγ. Data represent mean ± SD of biological duplicates from one healthy donor. Similar results were obtained for > 4 healthy donors. (E) LYC-53772 decreased the percentage of FOXP3^+^ cells during differentiation of murine Treg cells. *p = 1.6 × 10^−7^ LYC-53772 vs. Vehicle. (F) LYC-54143 decreased FOXP3^+^ cells during Type 17 differentiation of human PBMCs. *p = 0.009, LYC-54143 vs. Vehicle. Data represent mean ± SD of biological triplicates from one healthy donor. Similar results were obtained for three healthy donors. (G) LYC-54143 decreased the percentage of dead murine Type 17 T cells after differentiation and resting for 3 days. *p = 0.0001, LYC-54143 vs. Vehicle. Data represent mean ± SD of biological triplicates. LYC-53772 was used at 10 µM and LYC-54143 at 5 µM.

To assess whether synthetic agonists can enhance Type 17 differentiation, we tested the effects of LYC-53772 on murine Th17 and Tc17 differentiation. Splenocytes from OT-I (for Tc17) and OT-II (for Th17) mice were cultured in the presence/absence of LYC-53772 with OVA-derived peptides SIINFEKL or ISQAVHAAHAEINEAGR, respectively, and the polarizing cytokines TGFβ and IL-6 for 4 days. Signature cytokines from these cells were analyzed by ELISA and results are shown in Figs. 2A and B. When LYC-53772 was present during Th17 or Tc17 differentiation, levels of secreted IL-17A, IL-17F, and GM-CSF were significantly enhanced. IL-22 was also increased during Th17 differentiation. Tc17 cells, however, did not produce detectable levels of secreted IL-22 under these conditions. Similar effects were observed when mRNA levels of these cytokines were examined and an increase of IL-22 was detected in both Th17 and Tc17 cells (Fig. S2A). The extent of Type 17 differentiation on day 4 was assessed using intracellular staining. LYC-53772 significantly increased the percentage of CD4^+^ and CD8^+^ T cells that express IL-17A (from 12.0% to 20.0% for CD4^+^ and 21.4 to 40.4% for CD8^+^ T cells). Importantly, RORγ agonists have minimal impact on the expression of the key antitumor cytokine, IFNγ particularly in Tc17 cells (Fig. 2B). These data confirm that RORγ agonists enhance Type 17 cell differentiation.

To confirm that the effects of RORγ agonists were specific, splenocytes from C57/BL6 wild type or RORγ (−/−) mice were activated by plate bound anti-CD3 and soluble anti-CD28 antibodies, and polarized with TGFβ and IL-6 in the absence or presence of RORγ agonist LYC-54143. Under these differentiation conditions, elevated IL-17A expression was observed in wild-type cells treated with LYC-54143, while cells from RORγ (−/−) mice did not produce IL-17A and LYC-54143 did not increase IL-17A production (Fig. 2C), validating that the effects of our compounds are mediated by RORγ. In addition, when LYC-54143 was added in Th1 polarization conditions, no IL-17A production was detected and IFNγ was not significantly changed by LYC-54143 treatment (Fig. S2B), suggesting that LYC-54143 selectively modulates signature cytokines in RORγt-expressing Th17 cells.

To test the effects of RORγ agonists on primary human T cells, PBMCs were activated with anti-CD3/28 beads and differentiated under Th17 polarization conditions. IL-17A, IL-17F, and IL-22 were all increased by LYC-54143 (Fig. 2D). This effect was specific for type 17 cytokines only as IFNγ, a signature type 1 cytokine, was not affected by LYC-54143.

Th17 and Treg cells share similar differentiation requirements and their respective transcription factors, RORγt and FOXP3 functionally antagonize each other.^17,18^ Thus, we hypothesized that activating RORγ would limit Treg differentiation. To test this hypothesis, OT-II splenocytes were differentiated into Treg cells in the presence of TGFβ and IL-2, with or without LYC-53772. As shown in Fig. 2E, the percentage of cells expressing FOXP3 declined significantly from 17% to 6% when treated with the agonist LYC-53772 (Fig. 2E). A reduction of FOXP3 mRNA and an increase of IL-17A mRNA was also observed (Fig. S2C). Similarly, when natural Tregs were removed from human PBMCs and the remaining cells differentiated into FOXP3 expressing cells, the percentages of newly differentiated FOXP3^+^ cells was also reduced in the presence of RORγ agonist LYC-54143 (Fig. 2F).

Type 17 cells are long-lived with a stem-like molecular signature.^10,15^ Thus, we predicted that RORγ agonists would improve the survival of Type 17 cells. To test this hypothesis, we differentiated OT-II splenocytes into Th17 cells in the absence or presence of LYC-54143, rested the differentiated cells for 3 days and monitored cell death using 7-AAD by flow cytometry. As shown in Fig. 2G, significantly fewer dead cells were found in the LYC-54143 treated cells, supporting that RORγ agonists improve survival of Type 17 cells.

Overall, activation of primary T cells in the presence of a synthetic RORγ agonist enhances cytokine production, differentiation, and survival of Type 17 T cells and inhibits the formation of FOXP3^+^ Treg cells. These effects require the presence of RORγ and are consistent with reported functions of RORγ. The enhanced Type 17 effector cells resulting from RORγ agonist treatment are more effective and long-lived, which may provide superior antitumor activity.

### Novel immune modulatory effects of synthetic RORγ agonists in murine Type 17 T cells

**Figure 3.**
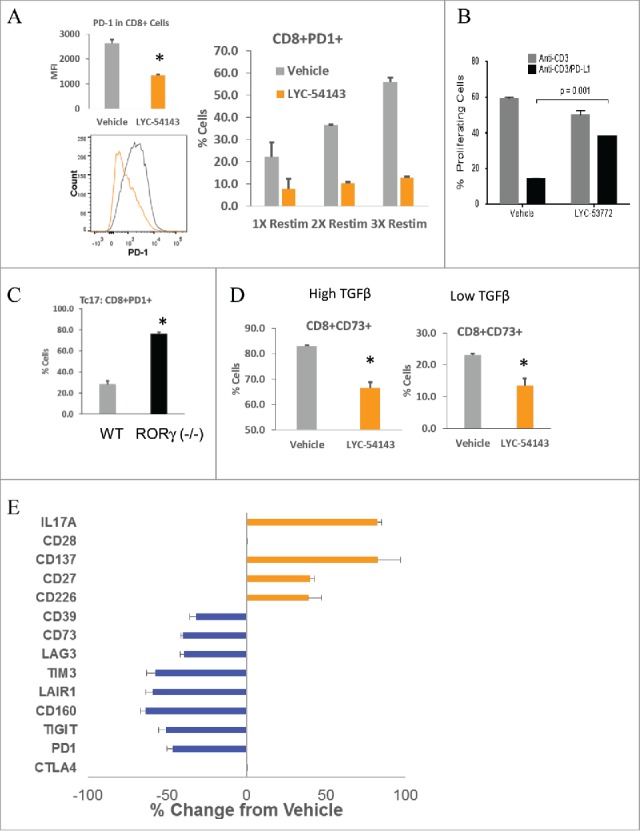
RORγ agonists modulate the expression of multiple co-regulatory molecules. (A) LYC-54143 decreased expression of PD-1 as well as percentage of PD-1^+^ cells in Tc17 cells. Left: MFI of PD-1 after one re-stimulation with anti-CD3. *p = 0.0002 LYC-54143 vs. Vehicle. Data represent mean ± SD of biological triplicates. A representative histogram is shown to compare vehicle (gray) with LYC-54143 (orange) treated samples. Right: percentage of PD-1^+^ cells after 1, 2, or 3 re-stimulations with anti-CD3. LYC-54143 was present during Tc17 differentiation but not during re-stimulation. (B) LYC-53772 counteracted the inhibitory effects of PD-L1. Proliferation of Type 17 T cells after re-stimulation with either anti-CD3 or anti-CD3/PD-L1 was analyzed using CFSE labeled cells. Data represent mean ± SD of biological duplicates. (C) Comparison of PD-1 expression in WT and RORγ deficient cells. *p = 0.00001 between WT and RORγ (−/−) cells. Data represent mean ± SD of biological triplicates. (D) LYC-54143 decreased the percentage of CD73^+^ cells in the presence of high (1.25 ng/mL) or low (0.25 ng/mL) concentrations of TGFβ. *p < 0.002 Vehicle vs. LYC-54143. Data represent mean ± SD of biological triplicates. (E) LYC-54143 increased the expression of multiple co-stimulatory receptors and decreased the expression of multiple co-inhibitory receptors in Type 17 T cells. The expression of indicated proteins was analyzed by flow cytometry. The frequencies of positive cells for each marker in vehicle and LYC-54143-treated cells were used to calculate % Change from Vehicle. Data represent mean ± SD of biological triplicates.

The recent success of immunotherapy with the immune checkpoint inhibitors anti-CTLA-4 and anti-PD-1/PD-L1 has demonstrated the critical roles of these receptors in suppressing antitumor immunity.^19,20^ Since Type 17 cells have been associated with enhanced antitumor immunity and RORγ is a transcription factor, we next examined if RORγ agonists could modulate the expression of PD-1. T cells isolated from C57/BL6 mouse spleens were subjected to Type 17 differentiation as described above in the presence/absence of LYC-54143. Differentiated cells were washed, rested, and re-stimulated with anti-CD3 monoclonal antibody to induce PD-1 expression and examined by flow cytometry. In both Tc17 (Fig. 3A) and Th17 (Fig. S3A) cell populations, a significant reduction of PD-1^+^ cells was observed when LYC-54143 was present during differentiation. When mean fluorescent intensity (MFI) was measured for the whole CD4^+^ or CD8^+^ population, there was also a significant reduction, suggesting that the RORγ agonist not only decreases the percentage of PD-1^+^ cells but also reduces the level of PD-1 on individual cells. The PD-1 suppressing effect of LYC-54143 was lost when T cells from RORγ (−/−) mice were used (Fig. S3B), suggesting an RORγ-dependent reduction. Moreover, the PD-1 level was much higher in RORγ (−/−) T cells (Fig. 3C) compared to wild-type Tc17 cells, further supporting a negative role of RORγ on PD-1 expression. Consistent with the requirement for RORγ to suppress PD-1, Tc0, or Tc1 cells which express very low levels of RORγ, have much higher PD-1 and no significant reduction of %PD-1^+^ cells was observed in these cells upon agonist treatment (Fig. S3C).

Elevated PD-1 in chronically activated T cells is a molecular signature of exhaustion.^21^ Interestingly, when differentiated Type 17 cells were repetitively re-stimulated with anti-CD3, PD-1 expression continued to increase after each round of stimulation, whereas cells treated with LYC-54143 only during initial differentiation maintained low levels of PD-1 after repetitive TCR stimulation (Fig. 3A). These results suggest that RORγ-agonist-induced effects are long lasting and resistant to repeated activation and may prevent T cell exhaustion. The reduction of PD-1 has functional consequences. When Type 17 cells were re-stimulated with anti-CD3 in the presence of PD-L1, the proliferation was significantly diminished. However, T cells treated with LYC-53772 were resistant to PD-L1 inhibition, resulting in restoration of their proliferative capacity (Fig. 3B and Fig. S3D).

Type 17 polarizing cytokines TGFβ and IL-6 induce the expression of ectonucleotidases CD73 and CD39, resulting in dampened antitumor effector functions.^22^ It was reported that Th17 cells differentiated with low concentrations of TGFβ together with IL-6 and IL-1β express lower levels of CD73 and better antitumor activity.^23^ LYC-54143 was assessed for its impact on CD73 expression under both differentiation conditions. Addition of LYC-54143 during Th17 polarization resulted in a significant reduction of CD73 under either high or low TGFβ conditions (Fig. 3D). The decrease of CD73 expression by LYC-54143 depends on the presence of RORγ (Fig. S3E). Similar reduction of CD39 was also observed (Fig. S3F).

Given the importance of co-inhibitory and co-stimulatory receptors in modulating immune responses,^19^ we also explored if RORγ agonists could regulate other co-inhibitory and co-stimulatory receptors implicated in antitumor immunity. The results are summarized in Fig. 3E. In addition to PD-1, exposing Type 17 cells to LYC-54143 during differentiation decreased the expression of the co-inhibitory receptors TIGIT, CD160, LAIR1, TIM3, and LAG3. On the other hand, LYC-54143 increased the expression of CD226, CD27, and CD137, co-stimulatory receptors that play important roles in antitumor immunity (Fig. 3E). LYC-54143 did not change the expression of CTLA4 or CD28 suggesting that only certain co-regulatory receptors are regulated by RORγ. Similar data were obtained using human T cells. Reduction in the percentage of CD4^+^ T cells expressing PD-1, CD73, CD160, or LAG3 by LYC-54143 was observed in differentiated human Type 17 T cells using PBMCs from cancer patients as well as healthy donors (Figs. S3G and H). Under our activation and differentiation conditions, no changes in percentages (all >90%) of cells expressing CD226 were observed. However, in most donors a small but significant increase in CD226 MFI was detected (Figs. S3G and H). These data suggest that the regulation of co-regulatory receptors by RORγ is largely conserved in human T cells.

Collectively, our data show that activating RORγ with a synthetic agonist, in a RORγ-dependent manner, decreases co-inhibitory receptor expression, diminishes expression of CD39 and CD73, enzymes important for generating immunosuppressive, extracellular adenosine, and increases co-stimulatory receptor expression. Taken together, these effects would be expected to drive more robust Type 17 effector immune responses and improve the potential for T cell-mediated tumor inhibition.

### Type 17 T cells treated with a synthetic RORγ agonist enhance cytotoxic activity in vitro and mediate potent antitumor immunity when adoptively transferred into mice with large tumors

**Figure 4.**
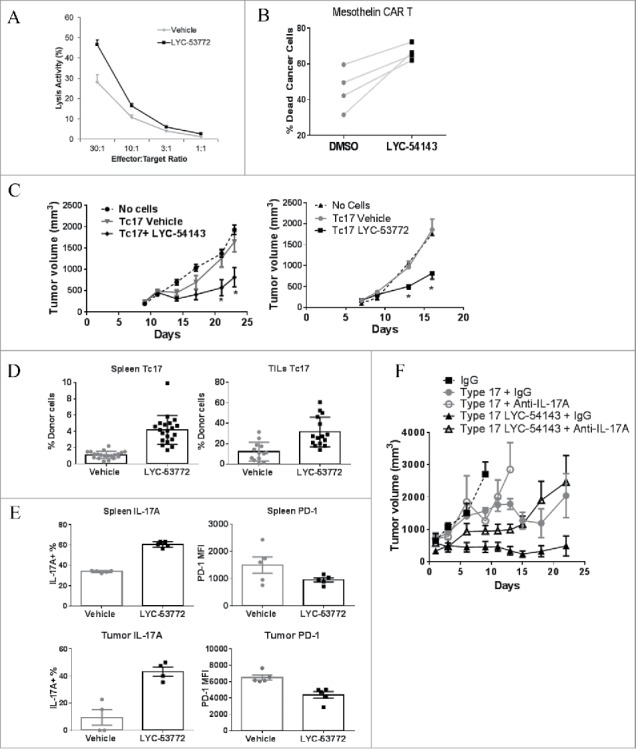
RORγ agonist treatment enhances cytotoxic activity of Tc17 cells *in vitro* and after transfer into tumor-bearing mice. (A) LYC-54143-treated Tc17 cells differentiated from OT-I T cells had better cytotoxic activity against EG7 lymphoma cells. *p* < 0.006 at all effector:target ratios LYC-54143 vs. Vehicle. Each data point represents mean ± SD of biological triplicates. B. LYC-54143 increased mesothelin CAR T mediated killing of K562.Meso tumor cells. *p* < 0.01, paired, two-tailed *t*-test. Differentiated Type 17 T cells from four donors treated with DMSO or LYC-54143 were transduced with a Meso-CAR construct and mixed with K562.Meso tumor cells. Killing of K562.Meso cells was assayed using flow cytometry. (C) Adoptive transfer of RORγ agonist treated Tc17 cells showed superior tumor growth inhibition in mice implanted with EG7 tumor cells. Left: LYC-54143 treated cells. *p < 0.025 LYC54143 vs. Vehicle. Multiple t-tests. Right: LYC-53772-treated cells. *p < 0.001 LYC53772 vs. Vehicle. Multiple t-tests. “No cells” group contains 10 mice and each of the Tc17-cell groups contains ≥12 mice. (D) Higher frequency of transferred donor cells were detected in the LYC-53772-treated group in both spleen and tumor at the end of the study. *p* < 0.0002 LYC-53772 vs. Vehicle. (E) LYC-53772-treated, adoptively transferred donor Tc17 cells from both spleen and tumor at the end of the study showed enhanced IL-17A production and reduced PD-1 expression. Percentage IL-17A^+^ cells among donor cells were assessed. PD-1 expression on donor cells was expressed as PD-1 MFI. LYC-53772 vs. Vehicle, *p* = 0.008 and 0.028, respectively, for IL-17A^+^ donor cells from spleen and TILs; *p* = 0.11 and 0.008, respectively, for PD-1 MFI in donor cells from spleen and TILs. (F) Neutralization of IL-17A with anti-IL-17A antibody reduced the antitumor efficacy of Type 17 cells treated with or without LYC-54143. Trp-1 and Pmel-1 T cells were differentiated into Type 17 cells and transferred into mice with B16 melanoma. IL-17A was neutralized by dosing anti-IL-17A antibody five times every other day starting from the day of transfer. N = 5 per group. *p* < 0.0001, Type 17 + IgG vs. Type 17 LYC-54143 + IgG; *p* = 0.0002, Type 17 LYC-54143 + IgG vs. Type 17 LYC-54143 + Anti-IL17A; *p* = 0.77, Type 17 + IgG vs. Type17 + Anti-IL17A and *p* = 0.37, Type 17 + Anti-IL17A vs. Type 17 LYC-54143 + Anti-IL17A. Mann–Whitney test.

Our data suggest that the changes induced by synthetic RORγ agonists in Type 17 T cells could be mechanistically linked to antitumor effector function. To test if RORγ agonists would enhance the tumor killing activity of cytotoxic Tc17 cells, OT-I Tc17 effector cells were generated in the presence or absence of LYC-54143, and then titrated onto CFSE-labeled, ovalbumin-expressing EG7 tumor cells. After 5 hours, the number of live EG7 tumor cells was quantified and the percent lysis was calculated. As shown in Fig. 4A, LYC-54143-treated Tc17 cells showed a significant increase in their ability to kill EG7 tumor cells at various effector:target ratios compared to untreated controls.

CAR T cell therapy has shown promising clinical efficacy in clinical trials.^24^ To assess if RORγ agonists could enhance cytotoxic activity of human cells, we utilized CAR engineered human T cells. Human CD4^+^ T cells were activated and polarized to a Th17 phenotype in the presence/absence of RORγ agonist LYC-54143, and then transduced with a CAR that recognizes mesothelin^25^ and expanded for 10 days. The resulting T cells were mixed with mesothelin-expressing tumor cells and tumor cell lysis was assayed by flow cytometry. As shown in Fig. 4B, LYC-54143-treated CAR T cells from four donors showed improved killing of tumor cells. These data support that RORγ agonists enhance the cytotoxic activity of human T cells and further suggest that RORγ agonists could be used to augment the tumor killing efficiency of CAR T cells when added during *ex vivo* expansion.

Having demonstrated that RORγ-agonist-treated Tc17 and CAR T cells showed enhanced tumor killing *in vitro*, we next asked if these cells could confer better tumor regression *in vivo* after being adoptively transferred into tumor-bearing mice. Thy1.1 OT-I CD8^+^ T cells were differentiated into Tc17 in the presence or absence of RORγ agonists and then equal numbers of cells were transferred into mice with established EG7 tumors. As shown in Fig. 4C, at the cell number used, Tc17 cells generated in the presence of vehicle had no significant effect on tumor growth compared with mice that did not receive tumor-specific T cells. However, when either LYC-53772- or LYC-54143-treated Tc17 cells were transferred, a significant inhibition of tumor growth was evident, confirming that RORγ agonists enhance the antitumor activity of Tc17 cells *in vivo*. At the end of the study, when spleens and tumors were examined, the number of transferred Thy1.1^+^ T cells was significantly higher in mice receiving RORγ-agonist-treated Tc17 cells compared with mice receiving vehicle-treated Tc17 cells (Fig. 4D and Fig. S4) despite equal numbers of cells being transferred, suggesting that the RORγ-agonist-treated cells survive and/or proliferate better than untreated cells after being transferred into tumor-bearing mice. Consistent with the *in vitro* cytokine data, the percentage of IL-17A-expressing cells among the donor cells was higher in the mice receiving LYC-53772-treated cells (Fig. 4E). In addition, donor Tc17 cells treated with LYC-53772 expressed less PD-1 in both spleen and tumor more than 2 weeks post-transfer, suggesting that the RORγ agonist exerted long-lasting effects on cytokine production and the expression of co-inhibitory receptors.

Given that enhanced IL-17A expression is maintained in agonist-treated T cells after adoptive cell transfer, we next asked whether IL-17A is required for the efficacy of agonist-treated cells.

CD4^+^ Trp-1 transgenic T cells and CD8^+^ Pmel-1 transgenic T cells can recognize tyrosinase-related protein 1 and gp-100, respectively, in B16 melanoma cells and have been used extensively in an adoptive cell transfer setting.^8,10^ When these transgenic T cells were differentiated under Type 17 polarization conditions in the presence/absence of LYC-54143, mixed at a 1:1 ratio and adoptively transferred into mice bearing B16 melanoma, a significantly better inhibition of B16 tumor growth was observed in mice receiving agonist-treated cells than mice receiving vehicle treated cells (Fig. 4F, Type 17 + IgG vs. Type 17 LYC-54143 + IgG). However, when IL-17A was neutralized using an anti-IL-17A antibody, tumor growth inhibition was significantly reduced in mice receiving LYC-54143-treated cells (Fig. 4F, Type 17 LYC-54143 + IgG vs. Type 17 LYC-54143 + Anti-IL-17A) relative to mice receiving vehicle-treated cells (Fig. 4F, Type 17 + IgG vs. Type 17 + Anti-IL-17A), suggesting that the antitumor efficacy of LYC-54143-treated cells is at least partially dependent on IL-17A in this model.

Together, these data indicate that *ex vivo* treatment of T cells with synthetic RORγ agonists induces durable changes that result in better tumor cell killing *in vitro* and following adoptive transfer. Collectively, our results provide a strong rationale for using RORγ agonists to improve ACT regimens.

### Oral administration of a synthetic RORγ agonist potentiates antitumor immunity

**Figure 5.**
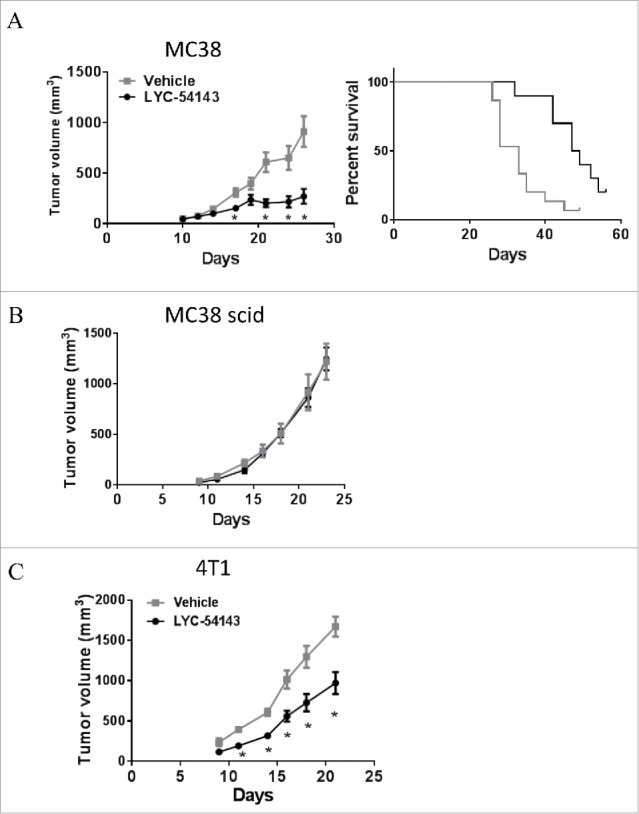
RORγ agonist LYC-54143 is efficacious in syngeneic tumor models. (A) LYC-54143 dosed twice daily at 100 mg/kg starting 3 days after subcutaneous tumor implantation, inhibited growth of MC38 colorectal tumor cells. Left: tumor growth curve *p < 0.01, LYC-54143 vs. Vehicle. N = 10 mice per group. Right: survival curve. Survival is defined as maintaining tumor size below ethical end points without significant weight loss (>20 %) or tumor ulceration. *p* = 0.0015 between the two groups, Log-rank (Mantel-Cox) test. (B) Efficacy is dependent on the presence of an intact immune system. No tumor growth inhibition with LYC-54143 treatment was observed when MC38 cells were implanted into SCID.Beige mice. N = 10 mice per group. (C) LYC-54143 was efficacious in the 4T1 breast cancer model. *p* < 0.004 LYC-54143 vs. Vehicle. N = 10 mice per group.

The data described above demonstrate that *in vitro* treatment of T cells with synthetic RORγ agonists enhance Type 17 effector functions directly. Thus, we hypothesized that *in vivo* administration of RORγ agonists to shape the developing antitumor immune response would be beneficial. To test this hypothesis, MC38 colorectal tumor cells were implanted subcutaneously into C57/BL6 mice and 3 days later, LYC-54143 was administered by oral gavage twice daily for 3 to 4 weeks. LYC-54143 was well tolerated and no signs of toxicity were observed. Tumor growth was significantly inhibited in mice receiving LYC-54143 (Fig. 5A, left). In addition, the inhibition of tumor growth translated into longer survival for the tumor-bearing mice compared to mice that received vehicle control (Fig. 5A, right). To investigate the involvement of immune cells, we implanted MC38 cells into SCID.beige mice that lack T, B, and NK cells. As shown in Fig. 5B, when LYC-54143 was administered to MC38-implanted SCID.beige mice, no tumor growth inhibition was observed. These results establish that the antitumor effects of RORγ agonist LYC-54143 are mediated by the immune system.

LYC-54143 was also tested in a second syngeneic tumor model, the 4T1 breast tumor model. This tumor model is resistant to many immunotherapies such as anti-PD-L1 or anti-CTLA-4 when administered as a single agent.^26,27^ Interestingly, LYC-54143 treatment resulted in significant growth inhibition of subcutaneous 4T1 tumors (Fig. 5C). These data suggest that RORγ agonists likely utilize overlapping but distinct mechanisms from anti-PD-1/PD-L1 or anti-CTLA-4 to control tumor growth in these syngeneic tumor models.

In conclusion, LYC-54143 is efficacious as a single-agent immunotherapy in multiple syngeneic tumor models. Thus, RORγ agonists offer a novel, promising immune therapy approach for the treatment of cancers.

## Discussion

To understand the roles of Type 17 cells in cancers, we focused on RORγ, the master transcription factor critical for differentiation of Type 17 cells. In various human cancers, CD3^+^CD4^+^RORγ^+^ cells are present at higher frequencies in the TILs than in PBMCs (Fig. 1A), suggesting a role for these cells in antitumor immunity. Similar findings were observed when CD3^+^CD4^+^IL-17A^+^ cells were assayed.^11^ However, it is important to note that although IL-17A is a direct target of RORγ, its expression is also regulated by other transcription factors such as Runx1, STAT3, and BATF.^28^ In addition, as a nuclear hormone receptor, RORγ activity is also influenced by endogenous ligands, which are tightly regulated^16^ and may not be present in some cells. These data are consistent with our observation that IL-17A and RORγ are not co-expressed in all T cells (Fig. 1B). Interestingly, a recent study showed that deficiency of IL-17A but not RORγt is associated with decreased spontaneous intestinal tumorigenesis in the APC^MIN/+^ mouse model,^29^ suggesting that IL-17A and RORγt may play distinct roles in cancers.

We used a chemical biology approach to explore if enhancing formation of Type 17 T cells with a synthetic ligand that activates RORγ could have effects on antitumor immunity. We identified potent, selective RORγ agonists which were confirmed to selectively increase RORγ target genes and enhance differentiation of Th17 and Tc17 cells (Fig. 2). Signature Type 17 cytokines IL-17A, IL-17F, IL-22, and GM-CSF, and chemokine CCL-20 were increased by agonist treatment. IFNγ, a critical cytokine for T cell effector function in tumors, was not significantly reduced by agonist treatment (Fig. 2B and Fig. S2B). These cytokines and chemokine have been shown to increase antitumor effector T cell functions in various tumor models. For example, IL-17A has been shown to inhibit tumor growth in immune competent mouse models via enhancing the generation of MHC-I and MHC-II antigens and tumor-specific cytotoxic cells.^30,31^ GM-CSF is known to promote formation of dendritic cells and cancer antigen presentation, and has shown clinical activity in cancer patients either as a systemic agent or vaccine adjuvant.^32,33^ Thus, a RORγ agonist could potentiate effector T cell functions through the action of these cytokines. Our preliminary data suggest that at least in the B16 adoptive transfer model, IL-17A is important for the antitumor effects of RORγ agonists (Fig. 4F). It will be interesting in future studies to evaluate what roles IL-17A and other cytokines play in mediating the antitumor effects of RORγ agonists across different tumor models.

Tumor cells escape immune surveillance by creating an immunosuppressive microenvironment, which includes the recruitment or generation of CD4^+^FOXP3^+^ Treg cells and/or conversion of effector T cells into Treg cells. It has been shown that the numbers of Treg and Th17 cells are inversely correlated in the tumor microenvironment.^11^ Consistent with the reported plasticity and reciprocal regulation of Treg and Th17 cells,^17,18^ enhancing Th17 differentiation with a RORγ agonist results in inhibition of Treg formation (Figs. 2E and F). Our results suggest that a RORγ agonist potentially can shift the balance to favor immune effector Th17 cells over immunosuppressive Treg cells. Notably, there exists a population of immunosuppressive T cells that co-express RORγ and FOXP3.^34,35^ The potential for RORγ agonists to reduce the immunosuppressive activity of these cells and convert them into Th17 effector cells will be interesting to explore in the context of cancer models.

T cells in the tumor microenvironment are frequently in an “exhausted” state exemplified by their reduced proliferation, cytokine production, and cytotoxic activity. These cells express multiple co-inhibitory receptors such as PD-1, CTLA-4, TIM3, and LAG3. The clinical successes of anti-PD-1 and anti-CTLA-4 suggest that overcoming T cell exhaustion represents a promising therapeutic approach to treat cancers. Here, we show that RORγ agonists reduce PD-1 expression in both Th17 and Tc17 cells (Fig. 3A). The negative regulation of PD-1 expression by RORγ is also supported by the higher PD-1 expression in RORγ deficient cells (Fig. 3C). In addition, human Th17 cells isolated from tumors have been reported to be almost devoid of PD-1 expression.^11^ Collectively, these data suggest that activation of RORγ suppresses PD-1 expression, which would be expected to mitigate PD-L1-mediated inhibition of antitumor responses. Moreover, the negative regulation of PD-1 by RORγ agonists is long lasting as RORγ-agonist-treated cells maintain reduced levels of PD-1 after repeated re-stimulation in the absence of an agonist compound and after being transferred into tumor-bearing mice, suggesting that the RORγ agonist may induce epigenetic changes in the PD-1 locus. In addition to PD-1, RORγ agonists also decrease several other co-inhibitory receptors that are hallmarks of T cell exhaustion. These receptors include TIM3, LAG3, TIGIT, CD160, and LAIR1.^36^ Blocking antibodies targeting these molecules are being developed for cancer treatment.^19,37^ Interestingly, one of these co-inhibitory receptors, TIGIT, shares ligands (PVR, PVRL2) with co-stimulatory receptor CD226.^38^ TIGIT inhibits while CD226 enhances cytotoxic activity of T cells and NK cells.^39-42^ Since we observed increased cytotoxic activity in agonist treated Tc17 cells (Figs. 4A and B), it is interesting to consider if RORγ agonist could potentiate cytotoxic activity via augmenting CD226 and at the same time reducing TIGIT expression.

In addition to CD226, RORγ agonists also enhance the expression of two other co-stimulatory receptors, CD27 and CD137 (Fig. 3E). These two co-stimulatory receptors belong to the tumor necrosis factor receptor superfamily (TNFRSF7 and TNFRSF9, respectively, for CD27 and CD137). Activation of these TNFRSF co-stimulatory receptors increases proliferation, survival, and effector functions of T cells.^38^ Agonistic antibodies that activate CD27 and CD137 are in clinical trials for various cancers with anti-CD137 showing promising clinical activity.^20,43^ The reciprocal regulation of co-inhibitory receptors and co-stimulatory receptors suggests that RORγ-agonist-treated cells may have a less immune suppressive and more immune active phenotype, which may be further reinforced by the reduced expression of CD39 and CD73, two enzymes that catalyze the formation of immunosuppressive adenosine from extracellular ATP and are being targeted for anticancer immunotherapy.^44^

Our results show that RORγ-agonist-treated Type 17 T cells produced higher levels of multiple inflammatory cytokines and expressed higher levels of co-stimulatory receptors. These immune stimulatory effects coupled with enhanced survival and/or self-renewal suggest that RORγ agonist treatment will induce durable immune activation. At the same time, RORγ agonist treatment limited Treg development, decreased immune checkpoint receptors and reduced extracellular adenosine formation, thus shifting the balance from immune suppression to immune activation. Collectively these effects translate into a better tumor killing activity both *in vitro* and following adoptive transfer into tumor-bearing animals.

Adoptive T cell therapy involves the *ex vivo* expansion of TILs or CAR T cells followed by infusion of the expanded T cells into patients. Despite early clinical success, many challenges still remain, including the lack of consistent engraftment and long-term survival of transferred T cells.^45^ Yet persistence of transferred T cells correlates with efficacy of ACT.^46^ In addition, under the influence of the immunosuppressive tumor microenvironment, transferred tumor-specific T cells can be converted to suppressive T cells such as Tregs or “exhausted” T cells.^36^ These challenges can dramatically limit the efficacy of ACT. We found that addition of a RORγ agonist enhances survival of Type 17 T cells and *in vitro* RORγ agonist treatment enhances the persistence of transferred T cells. In addition, RORγ agonist treatment limits the conversion of effector T cells into Treg and reduces T cell exhaustion markers to sustain the antitumor function of Type 17 T cells. These results suggest that RORγ agonists could be used as an adjuvant to ACT either by addition during the *ex vivo* expansion phase or by administration *in vivo* to augment the effector function and persistence of transferred cells for durable antitumor efficacy.

Using an orally bioavailable compound, LYC-54143, we demonstrated that activation of RORγ with a synthetic agonist induced robust antitumor effects in MC38 and 4T1 tumor models in an immune cell-dependent fashion (Fig. 5). These two tumor models have distinct TIL profiles, with abundant T cells in MC38 tumors and predominantly myeloid-derived suppressive cells (MDSCs) in 4T1 tumors. Various suppressive cells including Treg, MDSCs, and tumor-associated macrophages, are present in human tumors. Although we have not fully investigated the efficacy of RORγ agonists on tumors with various immune suppressive cells, our data suggest that RORγ agonists could be efficacious in tumors with diverse microenvironment, which is consistent with the ability of RORγ agonists to impact multiple antitumor mechanisms. Certainly, whether RORγ agonists could have therapeutic benefits will need to be determined in clinical trials with cancer patients.

Given that RORγ regulates many checkpoint receptors, we also speculate that a RORγ agonist will have synergistic or additive effects when combined with other immunotherapies, vaccines, radiation, targeted therapies, or chemotherapies. For instance, anti-CTLA-4 induces ICOS on CD4^+^ T cells.^47^ Co-stimulation via ICOS induces RORγt expression and enhances Th17 generation.^13^ Thus, combination of a RORγ agonist with anti-CTLA-4 may provide better efficacy than either single agent alone.

In summary, we show that RORγ agonists decrease immune checkpoint receptor expression, Treg generation, and extracellular adenosine generation while enhancing cytokine production, cytotoxic activity, and co-stimulatory receptor expression, and promoting the long-term survival and self-renewal of T cells. These results provide the rationale for testing a RORγ agonist in clinical settings as monotherapy or in combination with a checkpoint inhibitor such as anti-CTLA-4 or anti-PD-1. Overall, by integrating effects on multiple effector pathways, RORγ agonists represent a promising immunotherapy approach for the treatment of cancer.

## Materials and methods

### Reagents

All chemicals were purchased from Sigma, Avanti Polar Lipids, Tocris (R&D Systems) or Enzo Life Sciences. Antibodies used in flow analysis were purchased from eBioscience, Biolegend, or Miltenyi. RORγ agonists LYC-53772 and LYC-54143 were synthesized by Lycera. For further description of RORγ agonists and their use in cancer therapy, see, for example, international patent application publication WO 2015/131035.

### Cofactor recruitment assay and Luciferase reporter assay

These two assays were conducted as described previously.^16^

### Mouse type 17 cell differentiation

Splenocytes from OT-I or OT-II mice (Jackson Laboratories) were activated with OVA-derived peptides SIINFEKL (50 ng/mL) and ISQAVHAAHAEINEAGR (50 ng/mL), respectively, and polarizing cytokine TGFβ (1.25 ng/mL) and IL-6 (10 ng/mL) for 4 or 5 days. In low TGFβ condition, polarizing cytokines are TGFβ (0.25 ng/mL), IL-6 (10 ng/mL), and IL-1β (10 ng/mL). Un-polarized cells (Tc0 or Th0) were stimulated by the corresponding peptide.

Alternatively, splenocytes from C57/BL6 mice or RORγ(−/−) mice were activated with plate-bound anti-CD3 (2.5 μg/mL), soluble CD28 (0.5 μg/mL), and differentiated into Type 17 cells with either high TGFβ (1.25 ng/mL) and IL-6 (10 ng/mL), or Low TGFβ (0.25 ng/mL), IL-6 (10 ng/mL) and IL-1β (10 ng/mL). In some experiments, pan T cells were isolated from splenocytes using Pan T Cell Isolation kit (Miltenyi) and used in Type 17 cell differentiation. For Th1 and Th17 differentiation, naïve CD4^+^ T cells were isolated from splenocytes using naive CD4^+^ T Cell Isolation Kit (StemCell), activated by plate-bound anti-CD3 and soluble CD28 as above, and polarized into Th1 (10 ng/mL IL-12, 5 μg/mL anti-IL-4, and 1.25 ng/mL IL-2) or Th17 (1.25 ng/mL TGFβ and 10 ng/mL IL-6).

Treg cells were generated from OT-II splenocytes by OT-II peptide (20 ng/mL) together with TGFβ (5 ng/mL) and IL-2 (2.5 ng/mL) for 4 days. All cytokines used for differentiation were purchased from R&D systems.

IL-17A production was determined using mouse IL-17A ELISA (Mabtech). Type 17 differentiation was assessed by flow cytometer after 5 h of stimulation with PMA (100 ng/mL), Ionomycin (1 μg/mL) in the presence of brefeldin A (eBiosciences). Cell surface expression of co-stimulatory and co-stimulatory receptors was analyzed by staining with appropriate labeled antibodies (eBiosciences) directly. Intracellular staining was performed using labeled antibodies after fixation and permeabilization. Data were obtained using a BD FACSCanto II flow cytometer and analyzed by FACSDIVA software.

For Q-PCR analysis, RNA was isolated from differentiated cells by RNeasy mini kit (Qiagen) and mRNA expression was analyzed in StepOne Plus (Life Technologies) real-time PCR instrument using housekeeping gene β actin and cyclophilin as internal standards.

### Human type 17 cell differentiation

Human whole blood were obtained from healthy volunteers with written informed consent using protocols approved by Chesapeake Institutional Review Board.

PBMCs, isolated from whole blood using Ficoll centrifugation, were activated with anti-CD3/28 beads (Life Technologies) at 1:1 ratio and polarized into Type 17 cells with human IL-1β (10 ng/mL), IL-6 (10 ng/mL), and IL-23 (10 ng/mL). In some experiments, human recombinant TGFβ (0.5 ng/mL) were added to induce FOXP3 and CD73 expression. After 5 days, cytokine levels in the media were determined using a luminex panel (R&D Systems). Cells were collected for flow cytometry analysis.

PBMCs from cancer patients were purchased from Conversant Bio and activated with anti-CD3/28 beads and differentiated as described above.

### PD-L1 inhibition assay

Splenocytes were differentiated in Type 17 conditions as above. 5 days after differentiation cells were counted and rested overnight in fresh T cell growth media. Cells were then labeled with CFSE (ThermoFisher), stimulated with either anti-CD3/PDL-1-FC coated or anti-CD3/FC coated Dynabeads (M-450 tosylactivated) at a concentration of 1 bead/cell, plated at 0.5×10^6^ cells/mL in 96-well round bottom plates and incubated at 37°C and 5% CO_2_ for 7 days. Cell proliferation was analyzed by quantifying the fluorescent intensities of CFSE (cells with low CFSE peaks are proliferating cells).

### In vitro cytotoxicity assays

Tc17 cells were differentiated *in vitro* using total splenocytes from OT-I mice (C57BL/6-Tg(TcraTcrb)1100Mjb/J, Jackson Laboratory, #003831), with 50 ng/mL OT-I peptide (SIINFEKL) and 2.5 ng/mL TGFβ and 10 ng/mL IL-6. On day 4, Tc17 effector cells were washed and counted. E.G7-OVA cells were labeled with CFSE (10 µM) and counted. Various numbers of Tc17 effector (E) cells were mixed with E.G7-OVA target (T) cells in 96-round bottom plate, to achieve E:T ratio of 30:1, 10:1, 3:1 and 1:1. Mixed cells were incubated for 4 hours at 37°C with 5% CO_2_, and the lysis activity was calculated by E:T ratio at the end of the experiment.

### CAR generation and flow cytometry-based assay to quantify cytolysis

Blood samples were obtained from Pennsylvania Plasma. Peripheral blood CD4^+^ and CD8^+^ T cells were negatively isolated using an untouched T cell kits (Invitrogen) and cultured under Th17 conditions as previously described.^13^ For stimulation, T cells were cultured with activating beads coated with antibodies to CD3 and CD28 (eBioscience) at a 1:1 cell-to-bead ratio and then transduced with a Meso-CAR construct, as described.^13^ Target cells (K562 cells expressing mesothelin) were labeled with CFSE and seeded at 50,000 cells/well in 96-well plates. Human Th17/Tc17 cells primed or not with RORγ agonist LYC-54143 and CFSE-labeled target cells were co-cultured at 10:1 Effector:Target ratios for 8 hours. Total cells were stained with 7AAD and anti-CD45 antibody and analyzed on a flow cytometer.

### Animals

C57/BL6 and Balb/c mice were purchased from Charles River Laboratories. RORγ (−/−) mice were purchased from Jackson Laboratory. All animal experiments were conducted according to institutional animal care and safety guidelines and with IACUC approval at The University of Michigan (NCRC).

### Adoptive cell therapy tumor models

The EG7-OVA tumor cells (ATCC) are a cell line derived from a C57BL/6 lymphoma cell line EL4 which was engineered to express the neo-antigen ovalbumin. EG7 tumor cells were implanted subcutaneously into the flank of C57/BL6 mice and allowed to grow. In parallel, splenocytes from Thy1.1 OT-I mice were isolated and differentiated into Tc17 cells *in vitro* in the presence/absence of an RORγ agonist for 5 days. Once the tumor was measurable (normally between day 7 and 10 post-implant), the expanded T cells were injected intravenously or intraperitoneally. Antitumor responses were measured by assessing tumor volume over time. Tumor volume was assessed two to three times weekly using caliper measurement of length and width of tumor. Tumor volume calculation = 0.5 × (length × (width)^2^). Mice were taken down after tumor volume reached ethical end point of 2,000 mm^3^.

B16F10 melanoma cells were implanted subcutaneously into the flank of C57/BL6 mice. Trp-1 CD4^+^ cells and Pmel-1 CD8^+^ cells were harvested from TRP-1 and Pmel01 mice, differentiated into Type 17 cells in the presence/absence of LYC-54143 (10 µM) and transferred into B16F10 bearing mice as described.^8,10^ Neutralization of IL-17A were conducted as described.^10^

### Syngeneic tumor models

MC38 murine colon carcinoma cells or 4T1 murine breast carcinoma cells (ATCC) were implanted subcutaneously into the flank of C57/BL6 or Balb/c mice, respectively. Three days after implantation, mice were dosed with vehicle (1% Tween 80) or LYC-54143 at 100 mg/kg twice a day. Tumor volume, measurable 10–12 days after implantation, was assessed two to three time weekly using caliper measurement of length and width of tumor. Tumor volume calculation = 0.5 × (length × (width)^2^). SCID.beige mice (Jackson Laboratory) were also used as host mice for MC38 tumor cells to determine the immune system dependence. Mice were taken down after tumor volume reached ethical end point of 2,000 mm^3^.

### Data analysis and statistics

*In vitro* experiments were done with biological replicates higher than or equal to three unless otherwise noted in figure legends. Most critical experiments were conducted at least three times with similar results. Most data presented in figures are mean ± standard deviation (SD) of biological replicates. Statistics for *in vitro* data were done using unpaired, two-tailed *t*-test. Statistical comparison of treatment effects involving different human donors were analyzed using paired, two-tailed *t*-test. Statistics for *in vivo* data were done using Multiple *t*-tests, Mann–Whitney test or Log-rank (Mantel-Cox) test (survival curve) in GraphPad Prism 6.

## Supplementary Material

KONI_A_1254854_supplemental_material.pptx
