# Supplementary material for: Synthetic RORγ agonists regulate multiple pathways to enhance antitumor immunity
Source: Oncoimmunology. 2016 Nov 4;5(12):e1254854. doi: 10.1080/2162402X.2016.1254854 (PMC5215247; doi:10.1080/2162402X.2016.1254854)
Supplement: KONI_A_1254854_supplemental_material.pptx [file koni-05-12-1254854-s001.pptx]

## Slide 1
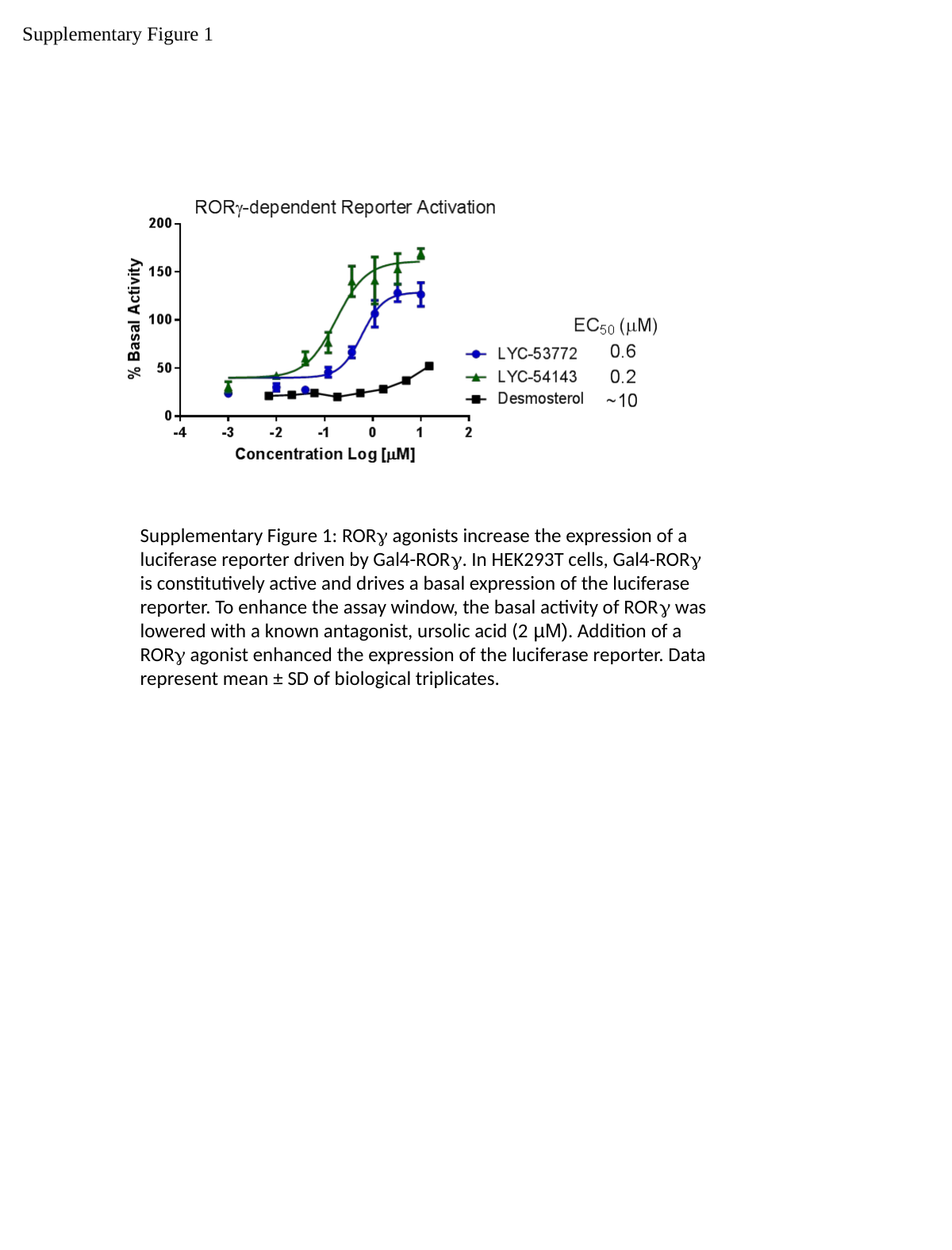

Supplementary Figure 1
Supplementary Figure 1: RORγ agonists increase the expression of a luciferase reporter driven by Gal4-RORγ. In HEK293T cells, Gal4-RORγ is constitutively active and drives a basal expression of the luciferase reporter. To enhance the assay window, the basal activity of RORγ was lowered with a known antagonist, ursolic acid (2 µM). Addition of a RORγ agonist enhanced the expression of the luciferase reporter. Data represent mean ± SD of biological triplicates.

## Slide 2
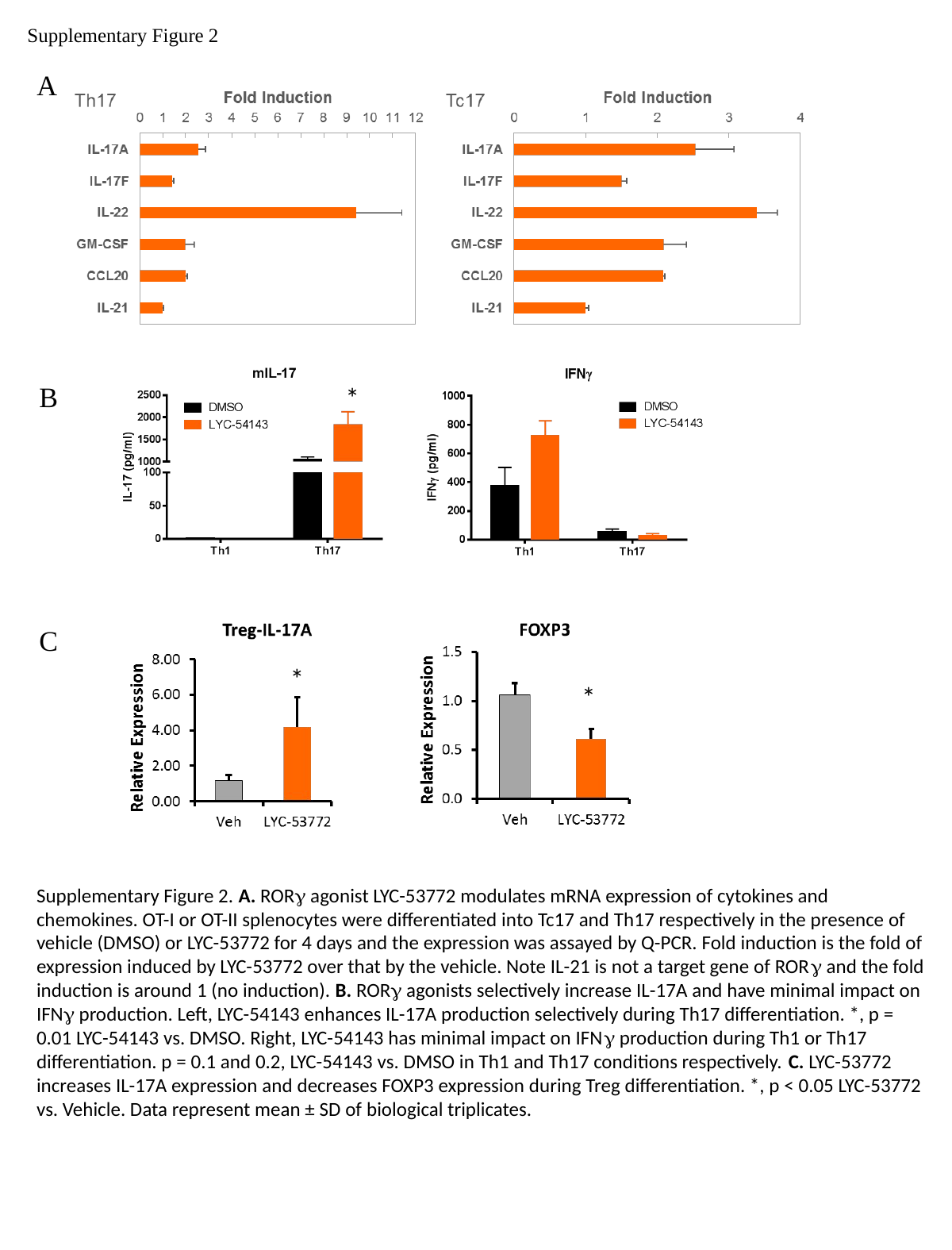

Supplementary Figure 2
A
B
*
C
*
*
Supplementary Figure 2. A. RORγ agonist LYC-53772 modulates mRNA expression of cytokines and chemokines. OT-I or OT-II splenocytes were differentiated into Tc17 and Th17 respectively in the presence of vehicle (DMSO) or LYC-53772 for 4 days and the expression was assayed by Q-PCR. Fold induction is the fold of expression induced by LYC-53772 over that by the vehicle. Note IL-21 is not a target gene of RORγ and the fold induction is around 1 (no induction). B. RORγ agonists selectively increase IL-17A and have minimal impact on IFNγ production. Left, LYC-54143 enhances IL-17A production selectively during Th17 differentiation. *, p = 0.01 LYC-54143 vs. DMSO. Right, LYC-54143 has minimal impact on IFNγ production during Th1 or Th17 differentiation. p = 0.1 and 0.2, LYC-54143 vs. DMSO in Th1 and Th17 conditions respectively. C. LYC-53772 increases IL-17A expression and decreases FOXP3 expression during Treg differentiation. *, p < 0.05 LYC-53772 vs. Vehicle. Data represent mean ± SD of biological triplicates.

## Slide 3
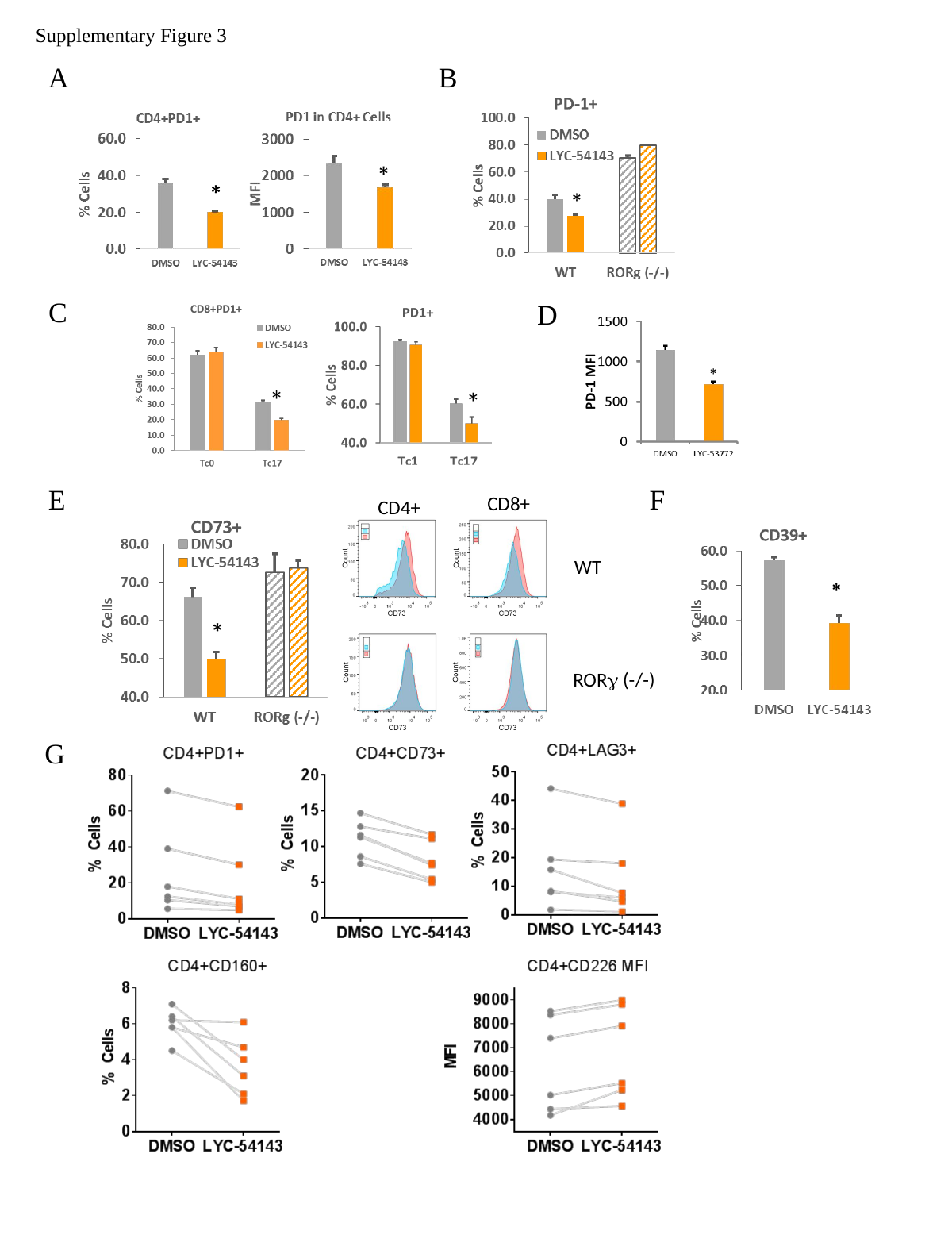

Supplementary Figure 3
A
B
C
D
*
*
E
F
CD8+
CD4+
WT
RORγ (-/-)
G

## Slide 4
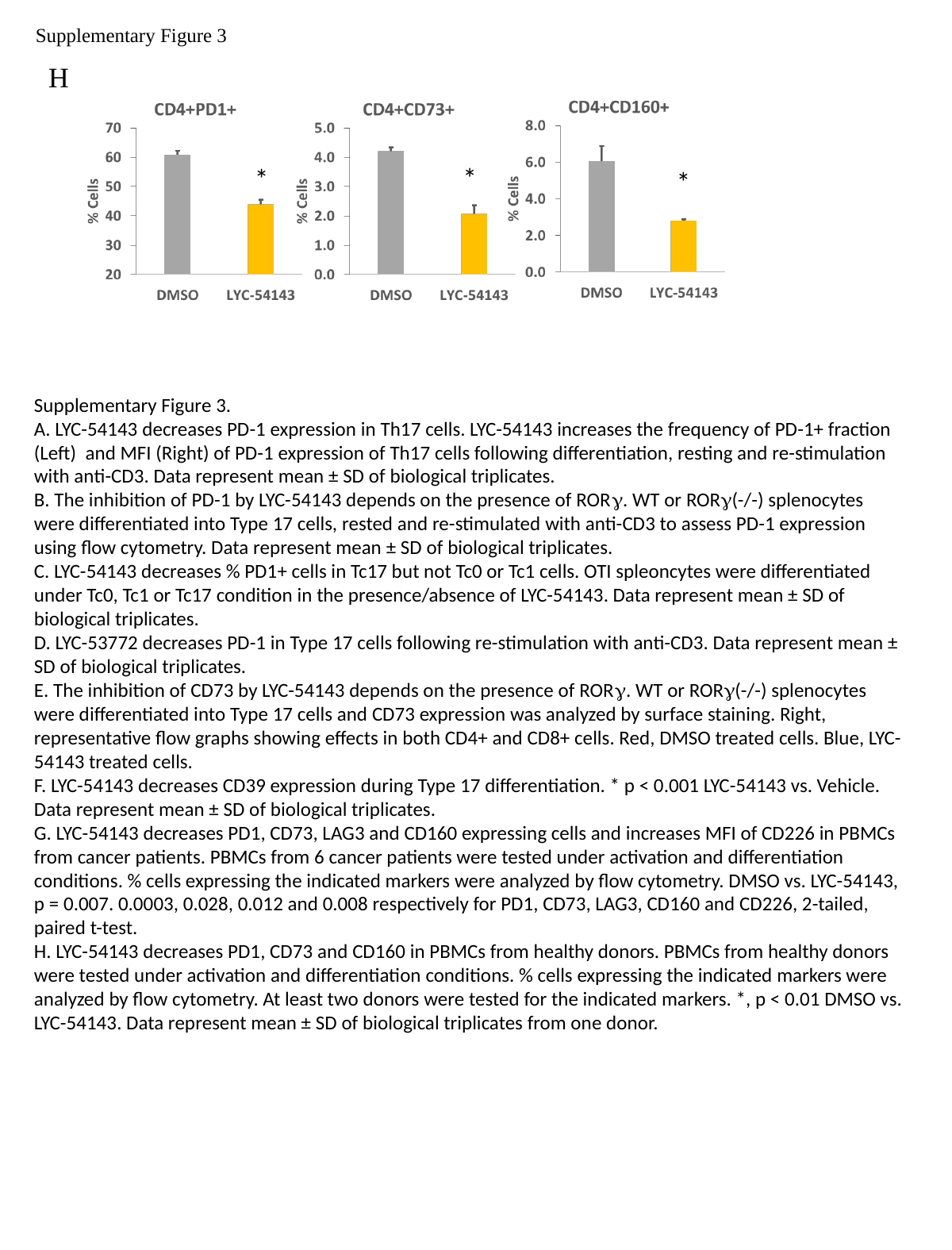

Supplementary Figure 3
H
*
*
*
Supplementary Figure 3.
A. LYC-54143 decreases PD-1 expression in Th17 cells. LYC-54143 increases the frequency of PD-1+ fraction (Left) and MFI (Right) of PD-1 expression of Th17 cells following differentiation, resting and re-stimulation with anti-CD3. Data represent mean ± SD of biological triplicates.
B. The inhibition of PD-1 by LYC-54143 depends on the presence of RORγ. WT or RORγ(-/-) splenocytes were differentiated into Type 17 cells, rested and re-stimulated with anti-CD3 to assess PD-1 expression using flow cytometry. Data represent mean ± SD of biological triplicates.
C. LYC-54143 decreases % PD1+ cells in Tc17 but not Tc0 or Tc1 cells. OTI spleoncytes were differentiated under Tc0, Tc1 or Tc17 condition in the presence/absence of LYC-54143. Data represent mean ± SD of biological triplicates.
D. LYC-53772 decreases PD-1 in Type 17 cells following re-stimulation with anti-CD3. Data represent mean ± SD of biological triplicates.
E. The inhibition of CD73 by LYC-54143 depends on the presence of RORγ. WT or RORγ(-/-) splenocytes were differentiated into Type 17 cells and CD73 expression was analyzed by surface staining. Right, representative flow graphs showing effects in both CD4+ and CD8+ cells. Red, DMSO treated cells. Blue, LYC-54143 treated cells.
F. LYC-54143 decreases CD39 expression during Type 17 differentiation. * p < 0.001 LYC-54143 vs. Vehicle. Data represent mean ± SD of biological triplicates.
G. LYC-54143 decreases PD1, CD73, LAG3 and CD160 expressing cells and increases MFI of CD226 in PBMCs from cancer patients. PBMCs from 6 cancer patients were tested under activation and differentiation conditions. % cells expressing the indicated markers were analyzed by flow cytometry. DMSO vs. LYC-54143, p = 0.007. 0.0003, 0.028, 0.012 and 0.008 respectively for PD1, CD73, LAG3, CD160 and CD226, 2-tailed, paired t-test.
H. LYC-54143 decreases PD1, CD73 and CD160 in PBMCs from healthy donors. PBMCs from healthy donors were tested under activation and differentiation conditions. % cells expressing the indicated markers were analyzed by flow cytometry. At least two donors were tested for the indicated markers. *, p < 0.01 DMSO vs. LYC-54143. Data represent mean ± SD of biological triplicates from one donor.

## Slide 5
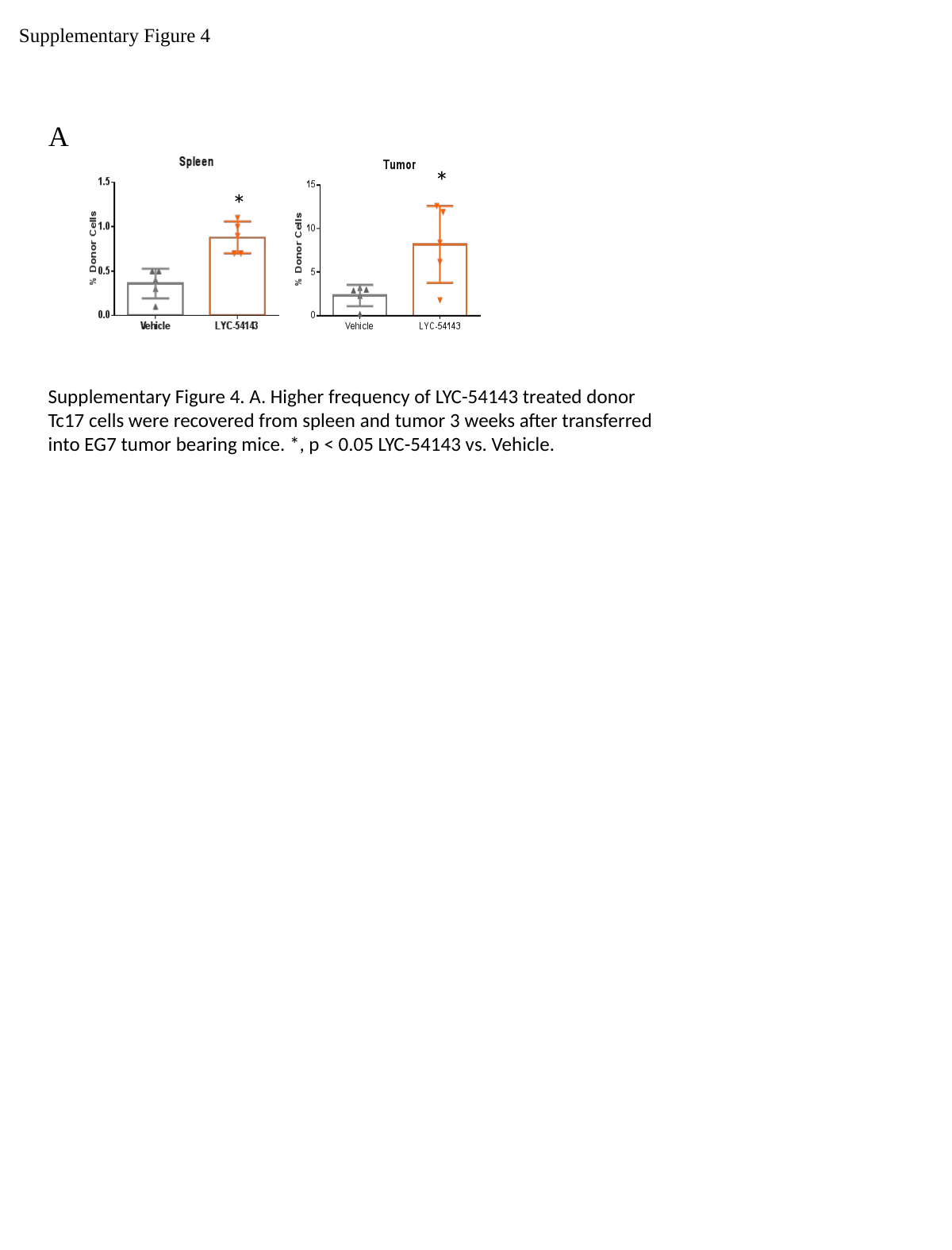

Supplementary Figure 4
A
*
*
Supplementary Figure 4. A. Higher frequency of LYC-54143 treated donor Tc17 cells were recovered from spleen and tumor 3 weeks after transferred into EG7 tumor bearing mice. *, p < 0.05 LYC-54143 vs. Vehicle.

## Slide 6
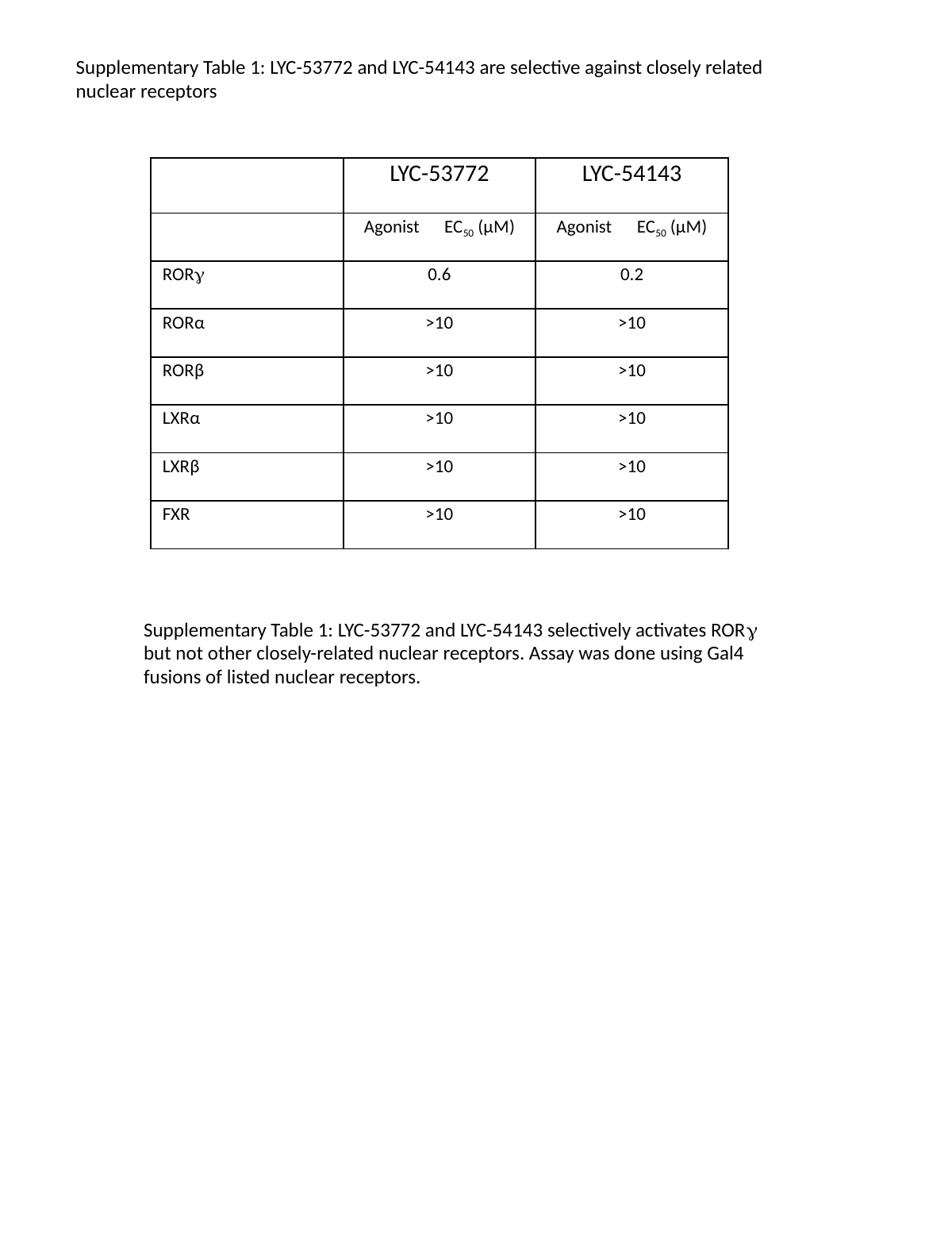

Supplementary Table 1: LYC-53772 and LYC-54143 are selective against closely related nuclear receptors
| | LYC-53772 | LYC-54143 |
| --- | --- | --- |
| | Agonist EC50 (µM) | Agonist EC50 (µM) |
| RORγ | 0.6 | 0.2 |
| RORα | >10 | >10 |
| RORβ | >10 | >10 |
| LXRα | >10 | >10 |
| LXRβ | >10 | >10 |
| FXR | >10 | >10 |
Supplementary Table 1: LYC-53772 and LYC-54143 selectively activates RORγ but not other closely-related nuclear receptors. Assay was done using Gal4 fusions of listed nuclear receptors.
